# Case reports on hand prejuvenation: clinical outcomes in middle-aged women with hyaluronic acid filler plus lidocaine

**DOI:** 10.1097/JW9.0000000000000185

**Published:** 2024-11-14

**Authors:** Gabriel Siquier-Dameto, Dennis Malvin Hernandez Malgapo

**Affiliations:** a Dameto Clinics International, Amsterdam, the Netherlands; b Dameto Clinics International, Campanet, Spain; c Research Group of Clinical Anatomy, Embryology and Neuroscience (NEOMA), Department of Medical Sciences, Universitat de Girona (UdG), Girona, Spain; d Skinsoul Advanced Dermatology and Medical Aesthetics, Cubao, Quezon City, Metro Manila, Philippines; e EW Villa Medica Manila, Newport Boulevard, Newport City, Metro Manila, Philippines

**Keywords:** future research direction (combination topical such as urea-based moisturizer or chemical peel skin resurfacing), hand prejuvenation (prevention aging and rejuvenation), reverse signs of aging, skin appearance, skin quality, younger-looking appearance

## Abstract

**Background:**

Hand rejuvenation with hyaluronic acid filler injections is gaining more attention for its ability to correct volume loss and improve skin quality. The hand dorsum is prone to loss of dermal elasticity and subcutaneous atrophy as individuals age, and administering hyaluronic acid fillers has become an option to restore the smoothness and youthful contour of the hand.

**Objective:**

The aim of this study is to demonstrate that injections with Definisse Touch Filler Plus Lidocaine are effective and safe for hand rejuvenation.

**Methods:**

Ten subjects underwent filler placement of 0.5 to 1.0 mL in the dorsal superficial lamina, which is devoid of sensory nerves and major skin vessels, using a blunt cannula via the fanning technique. Measurements were done before, immediately after, and 1 month after the treatment using validated aesthetic scales.

**Results:**

There was a significant correction of volume loss immediately after the first treatment and an improvement in skin texture and topography 1 month after the procedure. No lasting or severe adverse effects were observed.

**Limitations:**

To show significant and conclusive results, it is recommended to conduct further prospective, comparative, blinded studies in more subjects followed for longer periods (ie, 12 months or more). Objective analysis of volumetric changes using automated three-dimensional surface techniques is likewise beneficial.

**Conclusion:**

Injecting a low G-prime hyaluronic acid along the dorsal superficial lamina via cannula provides an effective and tolerable option for improving the appearance of aged hands.

**What is known about this subject in regard to women and their families?**

- The hands are among the most visible body parts. Many older women have a reduction in subcutaneous fat leading to the increased visibility of underlying bony prominences and tendons.
- Aged hands are a common indicator of age and have become a major cosmetic concern for many women who are concerned about their overall appearance.
- There are limited studies highlighting the use of filler material with lower hyaluronic acid concentration for hand rejuvenation. This study describes the clinical effects of small volumes of low hyaluronic acid filler material (ie, 23 mg/mL) for hand rejuvenation in a series of women subjects.

**What is new from this article as messages for women and their families?**

- Cosmetic enhancement of the hands may improve subjective well-being among women with prematurely aged hands.

## Introduction

The hands are subject to loss of dermal elasticity and atrophy of subcutaneous tissue as we age. Many factors contribute to these changes. For instance, our hands are exposed to ultraviolet (UV) radiation which is notorious for causing premature aging of the skin and deeper structures. Long-wave UVA, which penetrates deeper layers of the dermis, has a relatively milder impact on the superficial layers of the skin than short-wave UVB irradiation, which is mainly absorbed by the epidermis. Uncontrolled exposure to both UVA and UVB could lead to acute hyperesthesia and erythema. Chronic exposure can lead to extrinsic changes, which manifest as solar purpura, solar lentigines, and actinic keratoses. Irradiation with UVA can lead to intracellular changes, particularly DNA damage and alterations in gene expression, as well as inflammatory changes caused by the release of harmful reactive oxygen species in deeper areas of the hand.^1,2^ Finally, dermal and fat atrophy leads to volume loss exposing the underlying tendons, veins, and bony prominences, which may age the appearance of the dorsum of the hands. These changes occur at the dorsal superficial lamina (DSL), dorsal intermediate lamina, and dorsal deep lamina, the 3 fatty laminae of the hand.^3,4^ It is common to perform hand rejuvenation using filler material in 1 or more of these layers. Several types of subcutaneous fillers have been used for volumetric rejuvenation of the hands with varying treatment effects and longevity, including autologous fat, collagen, calcium hydroxyapatite, poly-L-lactic acid, and hyaluronic acid (HA). Autologous fat is biocompatible and may lead to potential neovascularization of injected tissue but is expensive and may be prone to side effects, such as infections in the areas of fat tissue harvest and uneven surfaces or lumps and prolonged edema over the injected areas. Porcine collagen has been tested in very few settings and may lead to uneven skin surfaces and potential hypersensitivity. The latter 3 filler types have all been shown to promote collagen formation but have the following limitations: calcium hydroxyapatite injected in boluses may be intimidating for patients, poly-L-lactic acid may require multiple treatments and a longer waiting period before seeing results leading to a higher cost of treatment, and HA fillers may lead to the Tyndall effect.^5^ More recent studies have focused on using subcutaneous HA filler injections to correct volume atrophy and superficial wrinkling and provide a smooth and youthful contour of the dorsal aspects of the hands^2^ as this material yields high patient satisfaction and has a relatively low risk for complications.^6^ Also, as shown in favorable anecdotal reports, HA filler injections of the hands represent a developing area in aesthetic surgery likely to gain popularity in the near future.^7^

The volumizing effect of HA fillers, in general, tends to decline over time, with most studies reporting a return to baseline after 12 months from the first injection.^7^ Definisse Touch Filler Plus Lidocaine (RELIFE S.r.l., Florence, Italy) is a subcutaneous cross-linked HA filler of 23 mg/mL HA with 0.3% of lidocaine used in the correction of moderate to deep wrinkles of the face and increase lip volume.^8^ A study of its rheology and physicochemical properties showed that this filler may be an option for patients who wish to have rejuvenating effects with a minimal amount of material.^8^ The typical volume of HA fillers used in studies on hand rejuvenation ranges from 0.5 mL to 12 mL with the need for the usual touch-up session to address persistent volume loss.^7^ The aim of this study is to demonstrate that injections in the dorsal surface of the hands with Definisse Touch Filler Plus Lidocaine could lead to effective and safe volumetric rejuvenation with a minimal amount of material. Minimizing the volume of filler material is expected to lower the risk of early and late complications associated with the introduction of foreign material into the skin and as a result, would make the treatment experience less daunting to patients.^9^

## Materials and methods

### Participants

All procedures performed in this study were in accordance with the ethical standards of Dameto Clinics International and with the Helsinki Declaration of 1975, as revised in 2008. Approval from an Institutional Review Board for studies on human subjects was not required because we have recorded and disclosed de-identified data. Informed, written consent was received from all patients from whom photographs have been derived. Between August and October 2020, one injector carried out the treatment in his private clinic and evaluated the rejuvenating effects of the procedure immediately and 1 month after the injections. Inclusion criteria included age ranging from 21 to 65 years, in good health (American Society of Anesthesiologists Classification 1), with physical signs of moderate to severe volume loss on the dorsum of the hands with a score of 1–4 according to the Global Aesthetic Improvement Scale (GAIS) score, a 5-point rating scale for assessment of changes in hand appearance. Exclusion criteria were recent previous fat grafts or fillers of the hand, systemic disease, psychiatric illness, a history of breast cancer surgery, allergy to HA or lidocaine, a history of arrhythmias (ie, Brugada syndrome), and pregnancy.^7^

### Treatment

The amount of Definisse Touch Filler with Lidocaine used in each injection varied depending on the severity of each case ranging from 0.5 to 1 mL per hand. Product placement was done through the DSL, which is a fatty layer deep into the skin lacking sensory nerves and major veins rendering it a suitable plane for filler injections.^10^ This was accomplished using a 25G × 38 mm tasukeru Steriglide cannula using the fanning technique. Injection techniques used for hand volume restoration in practice include tenting (injecting a single bolus by pinching the skin), serial puncture (a series of small volumes along a line), microdroplets (injecting minute amounts of filler at a large number of points), tunneling or linear retrograde threading (injecting the filler along a line in a retrograde fashion while withdrawing the needle), and fanning (without withdrawing the needle, several threads are injected radially).^5^ However, fanning was the preferred technique in this study as it limits the risk of bruising and minimizes the number of injection sites.

### Assessment

The subjects’ hands were evaluated for skin texture and quality before treatment using the validated Merz Aesthetic Scale (MAS), a 5-point photo-quantitative rating scale, and then for 5 to 10 minutes immediately after and for about 2 minutes at 1 month after treatment. Standardized digital photographs were likewise taken before, immediately after, and 1 month after the initial treatment. Adverse events were evaluated immediately after and at 1 month after treatment.

## Results

**Fig. 1.**
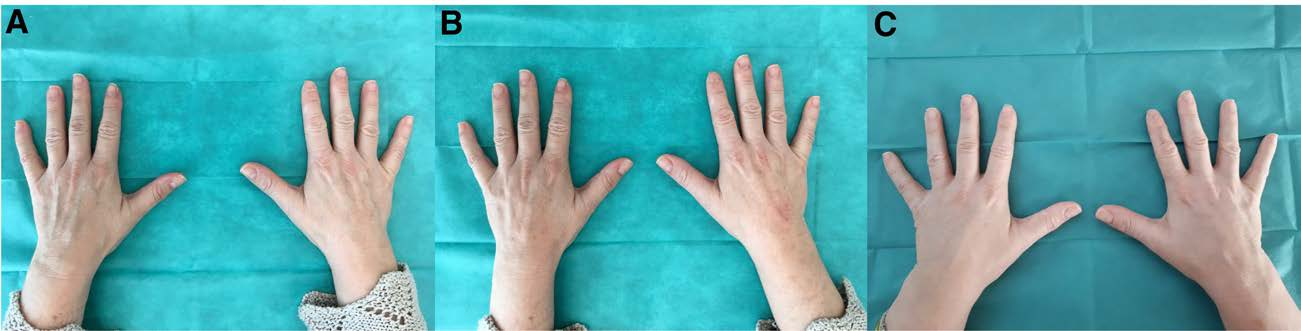
Pictures of patient 1 taken before treatment (A), immediately after treatment (B), and 1 month after treatment (C).

**Fig. 2.**
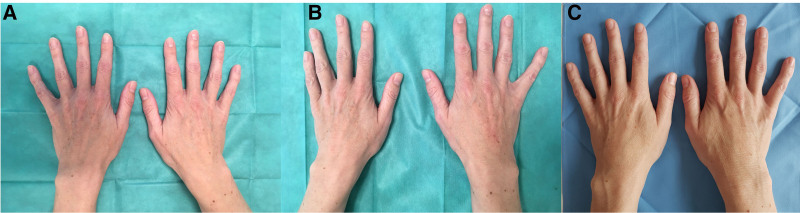
Pictures of patient 3 taken before treatment (A), immediately after treatment (B), and 1 month after treatment (C).

The subjects recruited in this study sought hand rejuvenation to correct volume loss and the overall aged look of the hand dorsum (Figs. 1A and 2A). Ten female subjects who were aged 35 to 55 years with a body mass index between 18.5 and 24.9 kg/m^2^ and in good health were enrolled in the study. There were no male study participants, a trend that is seen in other dorsal hand rejuvenation studies.^7^ All subjects underwent 1 treatment session without subsequent follow-up sessions. Four subjects required 0.5 mL per hand, while 6 necessitated up to 1.0 mL of filler injection.

Correction of volume loss was noticed immediately after the first treatment (Figs. 1B and 2B). One month after the procedure, skin texture and topography improved, as evidenced by the evenness of the hand dorsa in all subjects. Additionally, there was a reduction in the prominence of the dorsal veins of the hands. No apparent complications nor overcorrections were necessary immediately after the first injection and 1-month postinjection (Figs. 1C and 2C).

**Fig. 3.**
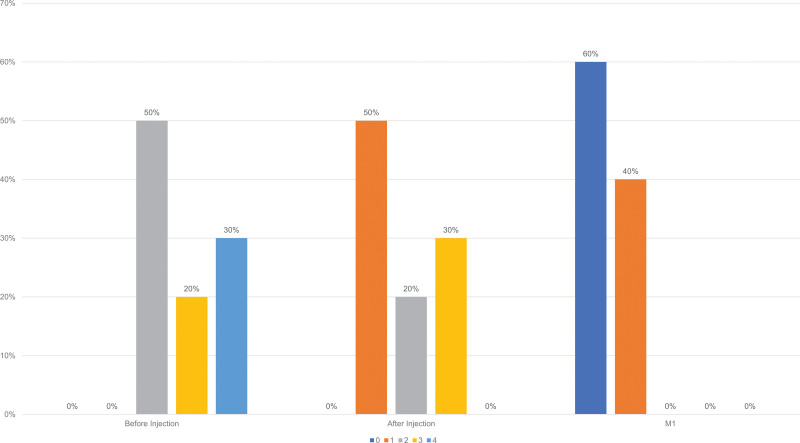
MAS scores from baseline, immediately after, and 1 month after treatment. MAS, Merz Aesthetic Scale.

The MAS scores were measured before, immediately after, and 1 month after the procedure. Zero denotes no loss of fatty tissue, while 1 to 4 means mild, moderate, severe, and very severe loss of fatty tissue, respectively, with gradually increasing visibility of veins and tendons.^11^ Patients with moderate to very severe MAS scores were observed to have had improved ratings immediately after and with progressive improvement at 1 month after treatment (Table 1). Baseline photographs revealed all patients experiencing moderate to very severe fat loss and mild to marked visibility of the dorsal hand veins and tendons. Immediately after treatment, most (70%) had grade 1 to 2 MAS scores (Table 2). Most patients had grade 0 to 1 MAS scores after 1 month of the procedure (Fig. 3), with an improvement in mean MAS scores of 0.4 from baseline 2.8 denoting marked improvement of hand appearance (Table 1).

**Table 1:** Merz Aesthetic Scale scores before, immediately after, and 1 month after treatment

Patient	MAS before treatment	MAS immediately after	MAS 1 month after
A	2	1	0
B	4	3	1
C	4	3	1
D	3	2	1
E	2	1	0
F	3	2	0
G	2	1	0
H	4	3	1
I	2	1	0
J	2	1	0
Mean MAS	2.8	1.8	0.4

MAS, Merz Aesthetic Scale.

**Table 2:** GAIS scores immediately after the first injection and at 1 month (“much improved” and “very much improved” have been pooled together into 1 category, “improved” is 1 category, and those rated with “no change” and worsened from baseline were pooled into another category)

	Immediately after (%)	1 month after (%)
Pooled “much improved” and “very much improved” scores	50	80
“Improved” scores	50	20
Pooled “no change” and worse from baseline scores	0	0

GAIS, Global Aesthetic Improvement Scale.

**Fig. 4.**
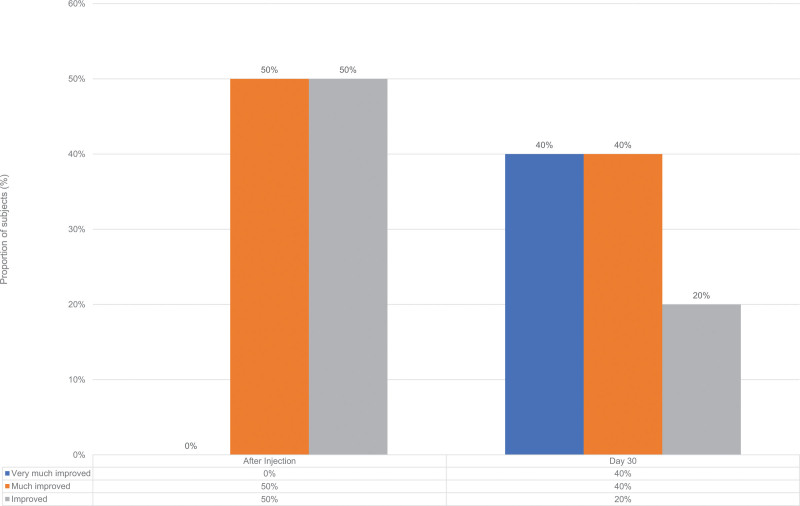
GAIS scores of patients undergoing hand rejuvenation with Definisse Touch Filler with Lidocaine immediately after the first injection and at 1 month. GAIS, Global Aesthetic Improvement Scale.

Likewise, the mean GAIS scores of the subjects were seen to have a general trend of improvement from 4 to 2.5 (Fig. 4). It can be observed that the enhancement of the subjective appearance of the hands persisted for 1 month, with up to 80% of patients experiencing “very much improved” and “much improved” appearance of their hands (Table 2).

## Discussion

Chronoaging results in the depletion of endogenous HA affecting the hydration, biomechanical integrity, and protection against oxidative stress of tissues in the dorsa of the hands. The introduction of HA fillers has been shown to clinically improve visible signs of volume loss and camouflage the tendons and veins^5,12^ through the improvement of the tissue microenvironment and the promotion of connective tissue and human fibroblast proliferation as shown in vivo.^13^

Visually, this can be seen as an improvement in dermal thickness and elasticity.^5,12^ In many cases, repeated injections with small to large amounts of fillers are needed to correct lingering volume deficits, and most studies report a return to baseline by 12 months after the first treatment.^7^ The administration of Definisse Touch Filler Plus Lidocaine led to photographic evidence of improvement in all subjects immediately after the first injection. When followed up after 1 month, the effects of the treatment persisted. The subjective GAIS scores of the patients showed a 100% improvement from baseline—a change that persisted among 100% of the subjects after 1 month of treatment and with a minimal amount of product. Likewise, MAS scores reflect investigator-observed improvement immediately after the injection and at 1 month following therapy.

This study highlights the importance of performing injections specifically within the DSL to avoid uneven correction and potential complications such as accidental injuries to dorsal veins, sensory nerves, or extensor tendons. Moreover, it demonstrated that the use of Definisse Touch Filler Plus Lidocaine can be used to achieve precise projection by using a very small amount of product applied to a single lamina. This filler exhibits viscoelastic properties that differ from other fillers with similar HA concentrations (ie, used for the same indications). It has a higher elastic/storage modulus (G’) (154 Pa) and viscous moduli (G’’) (26.67 Pa) than other low HA fillers, making it sufficient for hand rejuvenation in low amounts (0.5–1 mL) without the granularity associated with higher HA fillers.^8^ Finally, the treatment was found to be safe as there were no complications or side effects during the follow-up period.

## Limitations

This study is the first report of patients who have received Definisse Touch Filler Plus Lidocaine for volume correction of the hands. We acknowledge the limitations of this study. Prospective, comparative, blinded studies conducted in more subjects and in a longer timeframe (ie, 12 months or more) may be necessary to show significant and conclusive results.

Employing multiple blinded injectors and evaluators may improve the robustness of our results. Further, objective analysis of volumetric changes using automated three-dimensional surface techniques could have been helpful in objectively describing volumetric changes.

## Conclusions

Depending on its rheologic and physicochemical properties, minimal amounts of soft tissue fillers with low HA (ie, 23 mg/mL of HA in Definisse Touch Filler with Lidocaine) may provide effective volumetric correction of visibly aged hands. In this study, injecting HA along the DSL via a cannula has been shown to contribute to effective and well-tolerated HA filler administration immediately after and 1 month after treatment.

## Conflicts of interest

The authors made the following disclosures: G.S.D. and D.M.H.M. have consultancy contracts with RELIFE S.r.l.

## Funding

Supported by RELIFE S.r.l. in the form of an unrestricted educational grant.

## Study approval

This study was conducted with institutional approval from the ethics review board of Dameto Clinics International, following the principles of good clinical practice as outlined in ISO 14155:2020.

## Author contributions

GSD and DMHM equally contributed to the conceptualization, drafting of the research design, and data analysis. GSD recruited subjects and performed the interventions. GSD and DMHM drafted the manuscript and GSD reviewed it prior to submission.

## Patient consent

Informed, written consent was received from all patients for whom photographs are present in the manuscript.

## Acknowledgments

The authors would like to thank RELIFE S.r.l. for providing an unrestricted educational grant.
